# Sucralose Induces Biochemical Responses in *Daphnia magna*

**DOI:** 10.1371/journal.pone.0092771

**Published:** 2014-04-03

**Authors:** Ann-Kristin Eriksson Wiklund, Margaretha Adolfsson-Erici, Birgitta Liewenborg, Elena Gorokhova

**Affiliations:** Department of Applied Environmental Science, Stockholm University, Stockholm, Sweden; Federal University of Rio de Janeiro, Brazil

## Abstract

The intense artificial sweetener sucralose has no bioconcentration properties, and no adverse acute toxic effects have been observed in standard ecotoxicity tests, suggesting negligible environmental risk. However, significant feeding and behavioural alterations have been reported in non-standard tests using aquatic crustaceans, indicating possible sublethal effects. We hypothesized that these effects are related to alterations in acetylcholinesterase (AChE) and oxidative status in the exposed animals and investigated changes in AChE and oxidative biomarkers (oxygen radical absorbing capacity, ORAC, and lipid peroxidation, TBARS) in the crustacean *Daphnia magna* exposed to sucralose (0.0001–5 mg L^−1^). The sucralose concentration was a significant positive predictor for ORAC, TBARS and AChE in the daphnids. Moreover, the AChE response was linked to both oxidative biomarkers, with positive and negative relationships for TBARS and ORAC, respectively. These joint responses support our hypothesis and suggest that exposure to sucralose may induce neurological and oxidative mechanisms with potentially important consequences for animal behaviour and physiology.

## Introduction

The intense artificial sweetener sucralose is approved for human consumption in more than 70 countries. Due to the stability of the molecule, it is frequently found in recipient waters at concentrations ranging from 0.1 to 1.0 g L^−1^ [1]–[3], and has been detected even in offshore waters, such as the Atlantic Gulf Stream [4]. Due to this exceptional chemical stability in combination with high water solubility and widespread use, sucralose has been proposed as an ideal tracer for human activities [3]–[5]. Generally, it is not of concern as an environmental pollutant, and, indeed, sucralose has been shown to possess no bioconcentration properties [6], [7]. Also, no adverse acute toxic effects of sucralose have been observed using standard toxicity tests in aquatic plants, algae, crustaceans and fish [5], [7]–[9], and risk assessment propose a PEC/PNEC ratio well below 1, suggesting negligible risk to aquatic organisms [9]. However, in non-standard ecotoxicity tests, significant feeding and behavioural effects of exposure to sucralose were observed in crustaceans following a short term exposure [7], [10]. Due to this conflict of information, more studies on environmental fate and biological effects of this widespread environmental substance in non-target organisms are needed.

In an *in vitro* study using human liver cells, sucralose was found to react with cob(I)alamin, a reduced form of vitamin B_12_, forming alkylcobalamin (Suc-Cbl), and it was suggested that such reactions, may affect the cobalamin levels, analogous to exposure to epoxides and nitrous oxide [11]. Moreover, sucralose has raised concerns as a possible human health hazard, mostly in public media, because of its chlorinated structure [12], [13]. A chlorinated sugar, 6-Chloro-6 deoxyglucose, and its hydrolysis products that have structural resemblance with sucralose, have been shown to have degenerative effects on nerve cells [14], [15]. The responses to chlorosugars are very complex and species specific, varying greatly from no measurable effects to both sublethal (e.g., infertility) and acute (e.g., neurotoxicity) effects, depending on the chemical form of the sugar and the test organism [15], [16]. In crustaceans *Gammarus zadachi* and *Daphnia magna* exposed to sucralose, alterations in swimming behaviour were observed [7] raising concerns about its potential neurotoxic effects in aquatic animals. These macro- and microcrustaceans represent ecologically relevant groups in freshwater ecosystems and commonly used model species in ecotoxicology [17], [18]. “The Daphnia system” is becoming a leading research model for understanding environmental influences on various responses across different levels of biological organization and subsequent stressor induced acclimation and adaptation.

As behavioral aberrations observed in crustaceans may have a neurological origin, application of biomarkers to detect a neurological dysfunction would help understanding responses to environmentally relevant sucralose concentrations in these animals. Acetylcholinesterase (AChE) is an essential enzyme for the regulation of acetylcholine turnover responsible for terminating the transmission of many neuronal cell types across synapses. In (eco)toxicology, it is considered the most important biomarker of cholinergic signalling in the nervous system [19]–[21]. The AChE activity is thus a widely used biomarker [22], with the enzyme inhibition being a sign of chemically-induced neurotoxicity. However, increased AChE levels have also been observed in various species, including test organisms commonly used in ecotoxicology [23]–[25], which may be related to various roles of AChE responding to many external stimuli other than in cholinergic neurotransmission [26]. Moreover, evidence is accumulating that increased AChE activity observed in neurodegeneration is associated with high levels of reactive oxygen and nitrogen species (ROS and RNS, respectively) and oxidative stress. The latter is defined as an imbalance between endogenous free radical production through normal metabolism and antioxidant defences, and it is an important gateway to cellular damage caused by a variety of stress factors [27]–[28]. In particular, a direct linkage between oxidative stress and the enzymatic activity of AChE in the human brain has been suggested [29]. In ecotoxicological studies, the integrated assessment of AChE and oxidative stress biomarkers has also been advocated to increase sensitivity and understanding of the organismal effects observed in field and laboratory studies [30].

To our knowledge, this is the first study examining biomarker responses in aquatic organisms exposed to sucralose. Based on the observed swimming abnormalities in *Daphnia* exposed to sucralose [7] and recent findings that correlate AChE activity with oxidative stress in humans [29], [31], we hypothesized that these behavioural effects are related to alterations in AChE and oxidative status. More specifically, the following hypotheses were put forward: (1) alterations in AChE and oxidative status occur following exposure to sucralose; (2) lipid peroxidation and AChE responses to sucralose are modulated by antioxidants as antioxidative and prooxidative processes are intrinsically coupled; and (3) AChE responds in concert with oxidative damage measured as increase in lipid peroxidation levels. We tested these hypotheses experimentally by exposing *Daphnia magna* to a range of sucralose concentrations and measuring AChE activity in concert with commonly used biomarkers of oxidative status, oxygen radical absorbing capacity (ORAC; represents level of antioxidant defences) and lipid peroxidation (TBARS; thiobarbituric acid reactive substances, represents level of oxidative damage). Since oxidative and neurotoxicity biomarkers commonly exhibit non-monotonic or biphasic responses [32], [33], we applied generalized linear regression analysis (GLM) with an arbitrary function of the response variable (the link function) to vary linearly with the predicted values (rather than assuming that the response itself must vary linearly). As recently pointed out, GLM approach has a great potential in ecotoxicological studies, because it can enable us to explain several factors which could collectively contribute to the variation in a response variable (e.g., observed toxicity of the given chemical) or identify interrelationships between biomarkers, and, thus, provide a better understanding of the response mechanisms in ecologically relevant settings [34], [35]. The other advantages GLM in ecotoxicological studies, and particularly, mixed models with GLM, include unequal replicating, missed samples, flexible error distributions, etc. [36]; all these are highly appreciated when possibilities to have many standardized replicates within a treatment are limited. These advantages were particularly relevant in our study that was conducted using microscopic animals that provided very little material for biomarker analysis. Therefore, having a large number (several hundred) of individuals that could be used for the experiment and that are standardized in terms of age and feeding background was logistically challenging. To overcome this challenge, we run several consecutive experiments covering the broad range of sucralose concentrations (Table 1) and used GLM to account for possible variations between the experiments and differences in starting conditions of daphnia body.

**Table 1:** Experimental concentrations of sucralose and biomarker values measured in *Daphnia magna*.

Exp	Concentration	Protein	AChE	ORAC	TBARS
	mg L^−1^	µg ind^−1^	nmol min^−1^ µg^−1^	µg ind^−1^ trolox eqv.	pmol MDA ind^−1^
I	0.005; 0.05; 0.5	4.7–4.9	10.0–14.9	0.56–0.72	2.10–5.17
	Control	4.7	11.5	0.58	1.90
II	0.0005; 0.1	5.5–6.3	8.7–17.0	0.75–0.87	1.75–4.53
	Control	5.7	14.5	0.82	3.13
III	5	5.4–6.0	14.4–17.2	0.87	2.46–4.66
	Control	5.8	14.4	0.84	2.8
IV	0.001; 0.01; 1	4.6–5.5	6.8–17.0	0.41–0.71	1.68–5.10
	Control	5.0	9.8	0.64	3.39

A control using M7 medium was included in each of the four experiments (Exp I to IV). Data for the biomarkers within the experiment are shown as ranges (min-max) and for controls as mean values. Note that number of treatments differs among the experiments.

## Materials and Methods

### 1. Exposure of *Daphnia magna*

Neonates (<24 hour) of *Daphnia magna* were allowed to grow for three days in M7 medium and fed *Pseudokirchneriella subcapitata* following the recommendations of OECD Guideline 211. The 3-d old animals (Instars 2 and 3) were exposed to sucralose for 24 h in 100 mL jars, 30 individuals per beaker. As sucralose effects on mobility in *Daphnia magna* had previously been observed during a 24 h exposure [7], the subcellular level (biomarkers) were assumed to be detectable after this period. The sucralose concentrations tested were: 0.1, 0.5, 1, 5, 10, 50, 100, 500, 1000, and 5000 µg L^−1^; the M7 was used as a media; duplicate samples were obtained for each test concentration. Due to practical reasons, the test was conducted in four experimental runs. In each run, a batch of daphnids was distributed among the test concentrations and controls in two replicates. The range of the test concentrations in each experimental run is shown in Table 1; in each run, controls (M7 medium only) were used to normalize the treatment values to account for variations in the animal condition (e.g., body size) between the experiments. Upon termination of the exposure, the animals from each experimental jar were pooled into an Eppendorf vial (30 individuals sample^−1^; 2 samples per concentration) and stored at −80°C until the biochemical analyses. No mortality during the incubations was observed.

### 2. Sample preparation

For extraction, the samples were homogenized in 300 µL cold (4°C) potassium phosphate buffer (PPB; 0.1M, pH 7.2) [24] using MINI BEADBEATERS-8 homogenizer. The homogenate was centrifuged at 3300× g for 5 min at 4°C; the supernatant was collected, aliquoted and frozen in −80°C.

### 3. Biochemical assays

All biochemical analyses were performed using microplate reader FLUOstar Optima (BMG Lab Technologies, Germany) with absorbance and fluorescence configurations, depending on a particular assay. All samples, standards and blanks were analyzed in duplicates.

#### 3.1. BCA assay

Protein concentration (mg mL^−1^) was measured using the bicinchoninic acid assay (BCA, Pierce Ltd.) with bovine serum albumin (BSA) as standard according to the manufacturer's instructions. For each assay, 20 µL of the homogenate well^−1^ were used; the absorbance was measured at 540 nm, integration time of 1 s, 20 measurements well^−1^. The measured values were used to calculate individual protein weight of the test animals (µg ind^−1^).

#### 3.2. TBARS Assay

Lipid peroxidation was measured in185 µL homogenate mixed 1∶1 ice-cold trichloroacetic acid; PPB was used as a blank. The mixture was incubated on ice for 5 min and centrifuged at 9300× g for 5 min. Reaction solution (200 µL of 83 mM thiobarbituric acid (TBA) in glacial acetic acid: 1.5 M NaOH (1∶1) pH 3.5) was added to 200 µl of supernatant and incubated for 60 minutes in a boiling water bath. After cooling, 220 µL 1-butanol: pyridin (15∶1) mixture were added to all samples and standards, mixed for 2×10 s. Fluorescence was measured in the organic phase at excitation/emission wavelengths of 540/590 nm. Concentrations were derived from a standard curve of 1,1,3,3-tetramethoxypropane (malon-aldehyde acid; MDA; [37]). The results are reported in pM MDA equivalents ind^−1^.

#### 3.3. ORAC assay

The ORAC was measured using the modified ORAC_FL_ method [38] with fluorescein (Fluka; 79.6 nM well^−1^) as a fluorescent probe, 2,2′- azobis (2-amidinopropane) dihydrochloride (AAPH; Sigma–Aldrich; 23 mM well^−1^) as a peroxyl radical source, and Trolox (Sigma–Aldrich; 21.7 µM well^−1^) as a standard. For each assay, 4 µL of the homogenate were brought to 20 µL with PPB and used to measure ORAC; the values were expressed in trolox-equivalents, in µg ind.^−1^.

#### 3.4. AChE assay

The AChE activity was measured with the absorbance assay [39], [40] using acetylthiocholine iodide (AcSCh) as a substrate and dithiobisnitrobenzoate (DTNB) as a reagent. Specific activity was measured at 25°C and expressed as nmol of AcSCh min^−1^ mg protein^−1^.

### 4. Determination of sucralose in the exposure water

The sucralose concentrations were determined in duplicate samples representing controls and nominal concentrations of 5, 50, 500 and 5000 µgL^−1^; for logistical reasons, only 50% of all treatment samples and controls were analysed (Table S1 in File S1), which was considered acceptable given the low between-replicate variability in the analyzed samples. Sucralose (purity 98%) and sucralose-d6 (surrogate standard, isotopic purity >98%) were purchased from Toronto Research Chemicals Inc. (North York, Canada). Ammonium hydroxide 25% (puriss) was purchased from Fluka (Buchs, Switzerland). Methanol (LichroSolve) was purchased from Merck (Darmstadt, Germany). Water was of milli-Q grade from a milli-Q ultrapure water system, MilliQ PLUS 185 from Millipore (Stockholm, Sweden). Sucralose was extracted from the water samples by solid phase extraction [2]. Briefly, surrogate standard was added to 5 mL of water sample and sucralose was enriched on an Oasis HLB 60 mg 3 cc cartridge (Waters corp.). After elution with 2 mL methanol and change of solvent, the concentrations of sucralose were determined by electrospray LC/MS/MS.

### 5. Statistics

The duplicate experimental samples for each concentration were used as individual data-points in the regression analysis. All data were Box-Cox transformed to improve homogeneity and approach normal distribution. Generalized linear models (GLZ module) in STATISTICA 8.0 (StatSoft 2001, Tulsa, USA) with normal error function and log-link function were used to evaluate whether nominal sucralose concentration explained the variation in ORAC, TBARS, and AChE levels in the test animals. The level of variation in individual protein weight attributable to the experimental run and sucralose concentration was also estimated using GLZ to account for the between-run variability in animal condition; the variation of the latter is assumed to be represented by the variation in the individual protein weight (Table 1). All biomarker data were normalized to the respective control values in each experiment (Table 1). As ORAC levels were hypothesized to affect lipid peroxidation and AChE inhibition, their significance as covariables in the models for TBARS and AChE were tested. Also, the relationship between AChE and TBARS was evaluated to further test the involvement of oxidative mechanisms into the neurotoxic response. Finally, individual body size of daphnids measured as individual protein weight was initially included in all models; a biomarker: protein ratio was thus split into its components as this increases the sensitivity of the analysis [41]. Akaike Information Criterion (AIC) was used to optimise the number and combination of predictive variables included. To validate the proposed models, the Wald statistic was used to check the significance of the regression coefficient for each parameter, a likelihood ratio test was used to evaluate the statistical significance of including or not including each parameter and model goodness of fit was checked using deviance and Pearson χ2 statistics. Residual plots for each model were assessed visually to exclude remaining un-attributed structure indicative of a poor model fit. The relationship between nominal and measured sucralose concentrations was examined by ordinary linear regression.

## Results

The relationship between the nominal and measured sucralose concentrations was strongly significant (p<0.001, r^2^ = 0.99). However, the measured concentrations were approximately 40% lower than the nominal concentrations. Since only nominal concentrations were known for the entire dataset, they were used in the analysis of the experimental data; however, implications of the systematically overestimated exposure concentrations are believed to result in an underestimation of the real effects as addressed in the Discussion section.

There was a significant difference in protein weight among the sets of daphnids used in the different experiments (Table 1), with significantly higher values in the Experiment I (GLM; Wald = 5.814, df = 3, p<0.016; Table S2 in File S1). The variation in protein weight was not related to the exposure as indicated by no significant effect of the sucralose concentration (Wald = 1.064, df = 1, p>0.3).

The biomarker values varied both within and between the experiments (Table 1). When all the experimental data were considered, the biomarker variability was highest for TBARS, followed by AChE and ORAC (Table 1).

**Figure 1:**
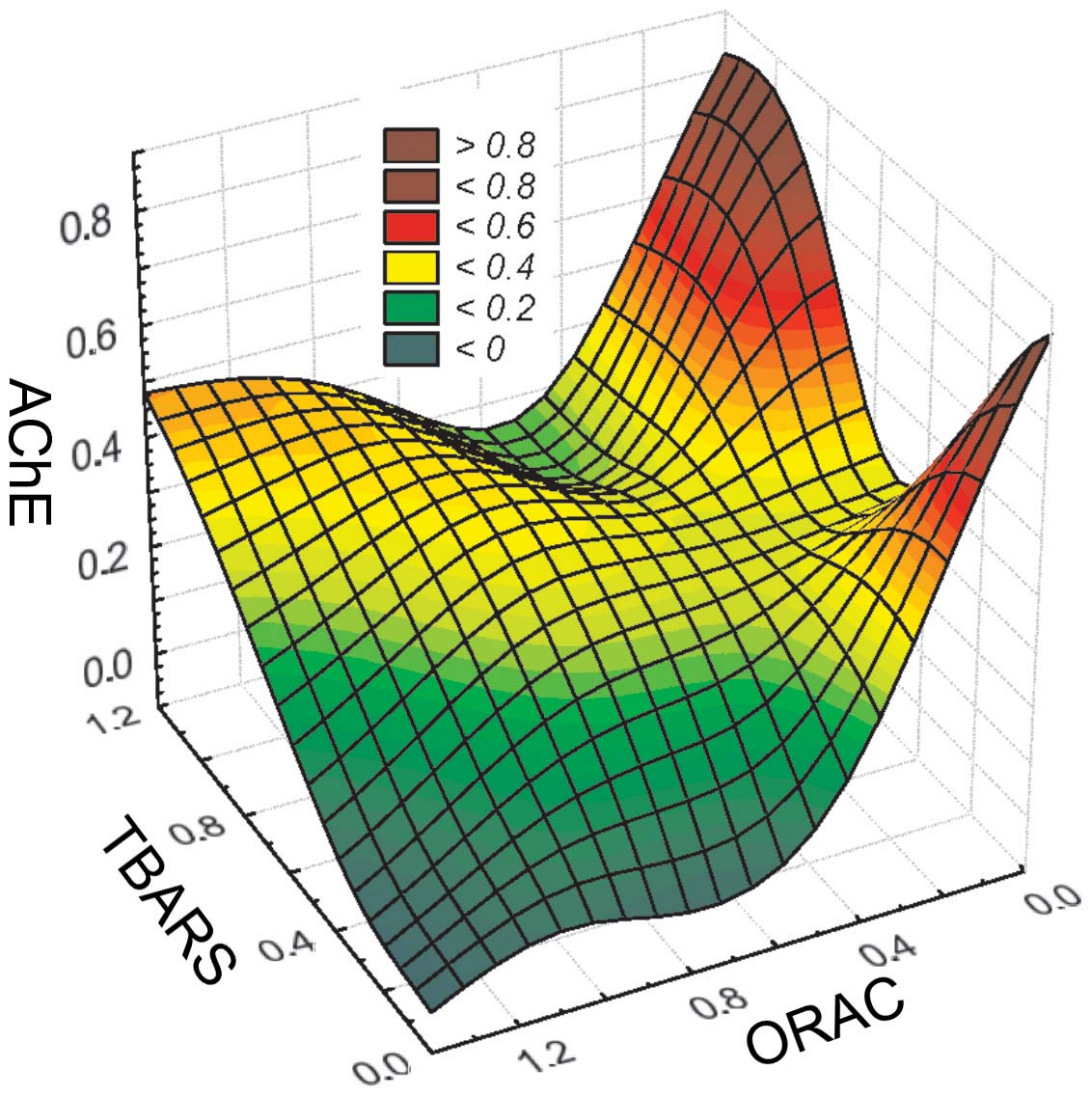
Relationships between AChE, ORAC and TBARS in *Daphnia magna* (all data from the Experiments 1–4 are combined). The data are Box-Cox transformed and normalized to the respective controls.

The nominal sucralose concentration was a significant positive predictor for TBARS, ORAC, and AChE activity in daphnids (Table 2), although none of the biomarkers followed a unidirectional concentration-dependent response. Moreover, normalized individual protein weight was a significant positive predictor for TBARS, with the largest effect size (Table 2). In the ORAC model, the protein weight, although not significant, had a large effect size and was retained as a covariable based on the AIC evaluation procedure (Table 2). The AChE response was linked to both oxidative biomarkers, with the positive and negative relationships with TBARS and ORAC, respectively (Table 2, Fig. 1). In the AChE model, the effect of ORAC was much stronger than that of the sucralose concentration (Table 2), whereas in the TBARS model, negative ORAC effect on lipid peroxidation levels was not significant, albeit retained in the model based on the AIC values (Table 2).

**Table 2:** Generalized Linear Models for biomarkers of oxidative damage (lipid peroxidation, TBARS, pmol ind^−1^), antioxidative defense (total oxygen radical absorbance capacity, ORAC, mg Trolox ind^−1^) and neurotoxicity (acetylcholinesterase activity, AChE, nmol AcSCh min^−1^ mg protein^−1^) in *Daphnia magna* exposed to a range of sucralose concentrations (sucralose, 0.1–5000 µg L^−1^); individual protein weight (protein, µg ind^−1^) was used as a proxy for body size.

Biomarker	Effect	Estimate	Standard error	Wald statistics	*p*
(A) TBARS	Sucralose	0.001	0.00	7.12	**0.008**
	Protein	4.243	2.15	3.91	**0.048**
	ORAC	−0.413	0.33	1.59	0.21
(B) ORAC	Sucralose	0.001	0.00	5.98	**0.01**
	Protein	2.54	2.23	1.30	0.25
(C) AChE	Sucralose	0.001	0.00	7.15	**0.007**
	ORAC	−0.249	0.78	10.31	**0.001**

(A) TBARS as a function of sucralose concentration, protein and ORAC; (B) ORAC as a function of sucralose concentration and protein; and (C) AChE as a function of sucralose concentration and ORAC. All data for the regression analysis were Box-Cox transformed; significant effects are in bold face.

## Discussion

We found a supporting evidence for the hypothesized effects of sucralose on biomarkers of neurotoxicity (AChE) and oxidative status (ORAC and TBARS) in the test species *Daphnia magna*. Moreover, these effects on AChE and TBARS were significantly ORAC-dependent, indicating that AChE activity and lipid peroxidation responded both directly to the sucralose exposure and indirectly to the alterations in the antioxidant levels. Also, the use of nominal concentrations in the statistical analyses may underestimate the real effect as the actual concentrations were considerably (∼40%) lower than the nominal concentrations. Although our experimental design suffered from other drawbacks, such as variations in daphnid body size between the experimental runs (which despite our efforts to account for that statistically may have introduced additional uncertainty) the overall co-variation in the biomarker responses indicates that alterations in AChE and oxidative status are interrelated. These coordinated responses further suggest that exposure to sucralose may induce neurological and oxidative mechanisms, with potentially important consequences for animal behaviour and physiology.

Inhibition of AChE activity is commonly interpreted as an indicator of neurological effects. However, we observed a stimulating effect of sucralose on the AChE activity. The reports of stimulatory effects on AChE activity are relatively scarce, but Holth and Tollefsen [42] found such effects on the electric eel *Electrophorus electricus* induced by the polar fraction of produced water from oil and gas production platforms. Moreover, algal toxins, metals and pesticides have been reported to induce a biphasic response in the AChE activity, with initial stimulatory effects followed by inhibition [43]–[45]. A similar time-dependent biphasic response to invariant concentration of a organophosphorous insecticide was found in the cladoceran *Bosmina* spp., with the initial stimulatory phase that coincided with increased mortality during the first 24 h of exposure and followed by AChE inhibition [46]. In humans, AChE activity generally increases with age [47]; furthermore, the elevated AChE activity has been linked to neurodegenerative diseases, e.g., Alzheimer's disease, Parkinson's disease, multiple sclerosis and Restless legs syndrome (RLS) [47], [48].

It has also been shown that in pathological conditions, oxidative mechanisms are involved in mediating effects on AChE [29]. In our study, AChE in sucralose-exposed *Daphnia* was negatively related to the antioxidant capacity measured as ORAC and positively to lipid peroxidation measured as TBARS, although the relationships were not linear (Table 2; Fig. 1). These observations are in line with several experimental studies linking AChE activity to oxidative stress in cell cultures and in vertebrate model organisms. For example, in cultured retinal cells, the increase in AChE activity induced by amyloid β-peptide was mediated by oxidative stress, indicating that antioxidants preventing the compromise of the enzyme activity have an important role in the maintenance of acetylcholine synaptic levels [29]. A study by Schallreuter and Elwary [49] showed that epidermal AChE is a target to H_2_O_2_-mediated oxidation of methionine and tryptophan residues and that this activation/deactivation of AChE by H_2_O_2_ is concentration-dependent. Interestingly, both AChE activity and lipid peroxidation increased in rats' brain as a result of exposure to cigarette smoke [50] and in zebrafish brain as a result of exposure to ethanol [51]. In line with this, we found that both biomarkers increased in response to sucralose exposure in *Daphnia magna* (Table 2; Fig. 1). It is well documented that the changes in AChE activity affect several physiological and behavioural processes and may have consequences for feeding, identification and avoidance of predators, and spatial orientation [52]. Similarly, changes in oxidative status are impinging on organism's fitness and may thus impact ecological interactions [53]. Indeed, the effects observed in daphnids that are ecologically important [54] as they are often the primary grazers of algae, bacteria and protozoans in freshwater systems and the primary forage for zooplanktivorous fish [55], may propagate in the food web and cause cascading responses.

Chemoreceptor studies in crustaceans have shown that carnivorous species detect primarily amino acids, while herbivorous and omnivorous species are also sensitive to carbohydrates [56]. Sugar receptors have been found in *Daphnia*, indicating that these animals likely sense some sugars, presumably dissolved in water and serving as cues for food sources [57]. In rats, however, significant differences in the taste perception for different sugars (i.e., maltose and sucrose) and a phenotypic variability in preference for sucralose have been reported [58], [59]. Therefore, it is possible that exposure to sucralose stimulates feeding and increases caloric intake that may also predispose the test animals to oxidative stress [60], [61]. Even though increased food intake might have occurred in our experiment, this has not translated into increased protein weight (i.e., growth) as indicated by no significant effect of sucralose concentration on the individual protein weight of *Daphnia* exposed to the broad interval of sucralose concentrations (Table S2 in File S1). In addition to the possible increase in food intake, some sugars (i.e., sucrose, lactose) can induce a heart arrhythmia in *Daphnia* [62]. Generally, feeding and heart rates change in concert, however, in cladocerans exposed to toxic algae, an asynchrony in these physiological responses during a short-term incubation has been observed, indicating a pathological condition that resulted in a long-term fitness decline [63]. More experimental studies are needed to measure coordinated physiological responses (feeding, metabolism and growth) to sucralose in animals with different diets in ecologically relevant settings as these effects are likely to be dependent on feeding habits and nutrition status of the test organisms.

A debate still ensues on the existence of biological effects of artificial sweeteners in non-target species living in areas receiving discharges from anthropogenic activities [3], [9]. Although sucralose was not found to bioaccumulate in the aquatic food chain from algae to daphnids and to fish, the bioconcentration factor (BCF) for the daphnids was slightly higher (BCF = 1.6–2.2) than for the algae and the fish (BCF<1) [6]. Whether this reflects a specific sensitivity of these grazers to the contaminant is unclear, but our results emphasize the importance of combining fitness-related responses with biomarkers and more generalized and ecologically relevant (grazing, food selectivity, energy transfer efficiency, etc.) *in situ* responses to identify ecological effects of environmentally relevant sucralose levels in surface waters.

## Supporting Information

File S1 **This file includes the following:** **Table S1.** Nominal and measured concentrations in the experiment. **Table S2.** Generalized linear model testing effects of the experimental run.
(DOCX)

## References

[1] Loos R, Gawlik BM, Boettcher K, Locoro GCS, Bidoglio G (2009) Sucralose screening in European surface waters using a solid-phase extraction-liquid chromatography-triple quadrupole mass spectrometry method. J Chromatogr 1216: 1126–1131. 10.1016/j.chroma.2008.12.048 19131070

[2] Minten J, Adolfsson-Erici M, Björlenius B, Alsberg T (2011) A method for the analysis of sucralose with electrospray LC/MS in recipient waters and in sewage effluent subjected to tertiary treatment technologies. Int J Environ Anal Chem 91: 357–366.

[3] Lange FT, Scheurer M, Brauch HJ (2012) Artificial sweeteners—a recently recognized class of emerging environmental contaminants: a review. Anal Bioanal Chem 403: 2503–2518. 22543693 10.1007/s00216-012-5892-z

[4] Mead RN, Morgan JB, Avery GB Jr, Kieber RJ, Kirk AM, et al (2009) Occurrence of the artificial sweetener sucralose in coastal and marine waters of the United States. Mar Chem 116: 13–17.

[5] Soh L, Connors KA, Brooks BW (2011) Fate of sucralose through environmental and water treatment processes and impact on plant indicator species. Environ Sci Technol 45: 1363–1369. 21235203 10.1021/es102719d

[6] Lillicrap A, Langford K, Tollefsen KE (2011) Bioconcentration of the intense sweetener sucralose in a multitrophic battery of aquatic organisms. Environ Toxicol Chem 30: 673–681. 21154846 10.1002/etc.433

[7] Wiklund AKE, Breitholtz M, Bengtsson BE, Adolfsson-Erici M (2012) Sucralose – an ecotoxicological challenger? Chemosphere 86: 50–55. 21955350 10.1016/j.chemosphere.2011.08.049

[8] Huggett DB, Stoddard KI (2011) Effects of the artificial sweetener sucralose on *Daphnia magna* and *Americamysis bahia* survival, growth and reproduction. Food Chem Toxicol 49: 2575–2579. 21742009 10.1016/j.fct.2011.06.073

[9] Tollefsen KE, Nizzetto L, Huggett DB (2012) Presence, fate and effects of the intense sweetener sucralose in the aquatic environment. Sci Tot Environ 438: 510–516. 10.1016/j.scitotenv.2012.08.060 23032567

[10] Hjorth M, Hansen JH, Camus L (2010) Short-term effects of sucralose on *Calanus finmarchicus* and *Calanus glacialis* in Disko Bay, Greenland. Chem Ecol 26: 385–393.

[11] Motwani HV, Qiu SR, Golding BT, Kylin H, Tornqvist M (2011) Cob(I)alamin reacts with sucralose to afford an alkylcobalamin: Relevance to in vivo cobalamin and sucralose interaction. Food Chem Toxicol 49: 750–757. 21130828 10.1016/j.fct.2010.11.037

[12] Abou-Donia MB, El-Masry EM, Abdel-Rahman AA, McLendon RE, Schiffman SS (2008) Splenda alters gut microflora and increases intestinal p-glycoprotein and cytochrome p-450 in male rats. J Toxicol Environ Health Part A 71: 1415–1429. 18800291 10.1080/15287390802328630

[13] Rodero AB, Rodero LD, Azoubel R (2009) Toxicity of sucralose in humans: a review. Int J Morphol 27: 239–244.

[14] Jacobs JM, Ford WCL (1981) The neurotoxicity and antifertility properties of 6-chloro-6-deoxyglucose in the mouse. Neurotoxicology 2: 405–417. 7199682

[15] Finn JP, Lord GH (2000) Neurotoxicity studies on sucralose and its hydrolysis products with special reference to histopathologic and ultrastructural changes. Food Chem Toxicol 38: S7–S17. 10882814 10.1016/s0278-6915(00)00024-7

[16] Viberg H, Fredriksson A (2011) Neonatal exposure to sucralose does not alter biochemical markers of neuronal development or adult behaviour. Nutrition 27: 81–85. 20116214 10.1016/j.nut.2009.10.007

[17] Kunz PY, Kienle C, Gerhardt A (2010) *Gammarus* spp. in aquatic ecotoxicology and water quality assessment: Toward Integrated Multilevel Tests. Rev Environ Contam Toxicol 205: 1–76. 20044794 10.1007/978-1-4419-5623-1_1

[18] Altshuler I, Demiri B, Xu S, Yan N, Cristescu ME (2011) An integrated multi-disciplinary approach for studying multiple stressors in freshwater ecosystems: *Daphnia* as a model organism. Integr Comp Biol 51: 623–633. 21873644 10.1093/icb/icr103

[19] Szabo G, Verma B, Fogarasi M, Catalano D (1992) Induction and modulation of transforming growth factor β and prostaglandin E2 by ethanol in human monocytes. J Leukocyte Biol 52: 602–611. 1464732 10.1002/jlb.52.6.602

[20] Behra M, Cousin X, Bertrand C, Vonesch JL, Biellmann D, et al (2002) Acetylcholinesterase is required for neuronal and muscular development in the zebrafish embryo. Nature Neurosci 5: 111–118. 11753420 10.1038/nn788

[21] Yang D, Howard A, Bruun D, Ajua-Alemanj M, Pickart C, et al (2008) Chlorpyrifos and chlorpyrifos-oxon inhibit axonal growth by interfering with the morphogenic activity of acetylcholinesterase. Toxicol Appl Pharmacol 228: 32–41. 18076960 10.1016/j.taap.2007.11.005 PMC2408880

[22] Tierney M, Nichols PD, Wheatley KE, Hindell MA (2008) Blood fatty acids indicate inter- and intra-annual variation in the diet of Adélie penguins: comparison with stomach content and stable isotope analysis. J Exp Mar Biol Ecol 367: 65–74.

[23] Srivatsan M (1999) Effects of organophosphates on cholinesterase activity and neurite regeneration in Aplysia. Chemico-Biol Interact 119–120: 371–378. 10.1016/s0009-2797(99)00048-4 10421473

[24] Jemec A, Drobne D, Tišler T, Trebše P (2007) The applicability of acetylcholinesterase and glutathione S-transferase in *Daphnia magna* toxicity test. Comp Biochem Physiol Part C: Toxicol Pharmacol 144: 303–09. 10.1016/j.cbpc.2006.10.002 17126609

[25] Gorokhova E, Löf M, Reutgard M, Lindström M, Sundelin B (2013) Exposure to contaminants exacerbates oxidative stress in amphipod *Monoporeia affinis* subjected to fluctuating hypoxia. Aquat Toxicol 127: 46–53. 22348951 10.1016/j.aquatox.2012.01.022

[26] Soreq H, Seidman S (2001) Acetylcholinesterase - new roles for an old actor. Nature Rev Neurosci 2: 294–302. 11283752 10.1038/35067589

[27] Das KC, White CW (2002) Redox systems of the cell: Possible links and implications. Proc Nat Acad Sci 99: 9617–9618. 12122214 10.1073/pnas.162369199 PMC124948

[28] Wilson SM, Gravel MA, Mackie TA, Willmore WG, Cooke SJ (2012) Oxidative stress associated with paternal care in smallmouth bass (*Micropterus dolomieu*). Comp Biochem Physiol Part A 162: 212–218. 10.1016/j.cbpa.2012.02.023 22414434

[29] Melo JB, Agostinho P, Oliveira CR (2003) Involvement of oxidative stress in the enhancement of acetylcholinesterase activity induced by amyloid beta-peptide. Neurosci Res 45: 117–127. 12507730 10.1016/s0168-0102(02)00201-8

[30] Lionetto MG, Caricato R, Giordano ME, Pascariello MF, Marinosci L, et al (2003) Integrated use of biomarkers (acetylcholinesterase and antioxidant enzymes activities) in *Mytilus galloprovincialis* and *Mullus barbatus* in an Italian coastal marine area. Mar Pollut Bull 46: 324–330. 12604066 10.1016/S0025-326X(02)00403-4

[31] Ranjbar A, Pasalar P, Abdollahi M (2002) Induction of oxidative stress and acetylcholinesterase inhibition in organophosphorous pesticide manufacturing workers. Hum Exp Toxicol 21: 179–182. 12099619 10.1191/0960327102ht238oa

[32] Wu H, Zhang R, Liu J, Guo Y, Ma E (2011) Effects of malathion and chlorpyrifos on acetylcholinesterase and antioxidant defense system in *Oxya chinensis* (Thunberg) (Orthoptera: Acrididae). Chemosphere 83: 599–604. 21194722 10.1016/j.chemosphere.2010.12.004

[33] Vandenberg LN, Colborn T, Hayes TB, Heindel JJ, Jacobs DR Jr, et al (2012) Hormones and Endocrine-Disrupting Chemicals: Low-Dose Effects and Nonmonotonic Dose Responses. Endocrine Rev 33: 378–485. 22419778 10.1210/er.2011-1050 PMC3365860

[34] Pokhrel LR, Dubey B (2013) Untangling Species Sensitivity Paradox in Environmental Research. Expert Opin Environ Biol 2: 1–2 Available: http://dx.doi.org/10.4172/2325-9655.1000e105. Accessed 2014 Feb 27.

[35] Glaholt SP, Chen CY, Demidenko E, Bugge DM, Folt C, et al (2012) Adaptive iterative design (AID): A novel approach for evaluating the interactive effects of multiple stressors on aquatic organisms. Sci Tot Environ 432: 57–64. 10.1016/j.scitotenv.2012.05.074 PMC3411930 22717606

[36] Dobson AJ, Barnett AG (2008) An introduction to Generalized Linear Models. 3rd ed. Chapman & Hall. 320pp.

[37] Shlafer M, Shepard BM (1984) A method to reduce interference by sucrose in the detection of thiobarbituric acid-reactive substances. Anal Biochem 137: 269–276. 6731812 10.1016/0003-2697(84)90084-8

[38] Prior RL, Hoang H, Gu L, Wu X, Bacchiocca M, et al (2003) Assays for hydrophilic and lipophilic antioxidant capacity (oxygen radical absorbance capacity (ORAC(FL))) of plasma and other biological and food samples. J Agric Food Chem 51: 3273–3279. 12744654 10.1021/jf0262256

[39] Ellman G, Courtney K, Andres V, Featherstone RM (1961) A new and rapid col-orimetric determination of acetylcholinesterase activity. Biochem Pharmacol 7: 88–95. 13726518 10.1016/0006-2952(61)90145-9

[40] Bocquené G, Galgani F (1998) Biological effect contaminants: cholinesterase inhibition by organophosphate and carbamate compounds. ICES Tech Mar Environ Sci 22: 1–12.

[41] Johannsson OE, Bowen KL, Arts MT, Smith RW (2009) Field assessment of condition indices (nucleic acid and protein) in *Mysis diluviana* . Aquat Biol 5: 249–262.

[42] Holth TF, Tollefsen KE (2012) Acetylcholine esterase inhibitors in effluents from oil production platforms in the North Sea. Aquat Toxicol 112–113: 92–98. 10.1016/j.aquatox.2011.10.019 22115844

[43] El-Merhibi A, Kumar A, Smeaton T (2004) Role of piperonyl butoxide in the toxicity of chlorpyrifos to *Ceriodaphnia dubia* and *Xenopus laevis* . Ecotox Environ Safe 57: 201–212. 10.1016/S0147-6513(03)00082-4 14759667

[44] Lehtonen KK, Kankaanpa H, Leinio S, Sipia VO, Pflugmacher S, et al (2003) Accumulation of nodularin-like compounds from the cyanobacterium *Nodularia spumigena* and changes in acetylcholinesterase activity in the clam *Macoma balthica* during short-term laboratory exposure. Aquat Toxicol 64: 461–476. 12878416 10.1016/s0166-445x(03)00101-2

[45] Bainy ACD, Gennari de Medeiros MH, Di Mascio P, Alves de Almeida E (2006) In vivo effects of metals on the acetylcholinesterase activity of the *Perna perna* mussel's digestive gland. Biotemas 19: 35–39.

[46] Sibley PK, Chappel MJ, George TK, Solomon KR, Liber K (2000) Integrating effects of stressors across levels of biological organization: examples using organophosphorus insecticide mixtures in field-level exposures. J Aquat Ecosyst Stress Recov 7: 117–130.

[47] Toiber D, Soreq H (2005) Cellular stress reactions as putative cholinergic links in Alzheimer's disease. Neurochem Res 30: 909–919. 16187225 10.1007/s11064-005-6963-8

[48] Akaike A, Takada-Takatori Y, Kume T, Izumi Y (2010) Mechanisms of Neuroprotective Effects of Nicotine and Acetylcholinesterase Inhibitors: Role of α4 and α7 Receptors in Neuroprotection. J Mol Neurosci 40: 211–216. 19714494 10.1007/s12031-009-9236-1

[49] Schallreuter K, Elwary S (2007) Hydrogen peroxide regulates the cholinergic signal in a concentration dependent manner. Life Sci 80: 2221–2226. 17335854 10.1016/j.lfs.2007.01.028

[50] Thomé G, Spanevello R, Mazzanti A, Fiorenza A, Duarte M, et al (2011) Vitamin E decreased the activity of acetylcholinesterase and level of lipid peroxidation in brain of rats exposed to aged and diluted sidestream smoke. Nicotine Tob Res 13: 1210–1219. 21896885 10.1093/ntr/ntr154

[51] Rosemberg D, Rocha R, Rico E, Zanotto-Filho A, Dias R, et al (2010) Taurine prevents enhancement of acetylcholinesterase activity induced by acute ethanol exposure and decreases the level of markers of oxidative stress in zebrafish brain. Neuroscience 171: 683–692. 20884336 10.1016/j.neuroscience.2010.09.030

[52] Pan G, Dutta HM (1998) The inhibition of brain acetylcholinesterase activity of juvenile largemouth bass *Micropterus salmoides* by sublethal concentrations of diazinon. Environ Res 79: 133–137. 9841812 10.1006/enrs.1998.3868

[53] Nussey DH, Pemberton JM, Pilkington JG, Blount JD (2009) Life history correlates of oxidative damage in a free-living mammal population. Funct Ecol 23: 809–817.

[54] Carpenter SR, Kitchell JF, Hodgson JR, Cochran PA, Elser JJ, et al (1987) Regulation of lake primary productivity by food web structure. Ecology 68: 1863–1876. 29357166 10.2307/1939878

[55] Tessier AJ, Leibold MA, Tsao J (2000) A fundamental trade-off in resource exploitation by *Daphnia* and consequences to plankton communities. Ecology 81: 826–841.

[56] Corotto FS, O'Brien MR (2002) Chemosensory stimuli for the walking legs of the crayfish *Procambarus clarkii* . J Chem Ecol 28: 1117–1130. 12184392 10.1023/a:1016273431866

[57] Peñalva-Arana DC, Lynch M, Robertson HM (2009) The chemoreceptor genes of the waterflea *Daphnia pulex*: many Grs but no Ors. Evol Biol 9: 1–11. 10.1186/1471-2148-9-79 PMC2680840 19383158

[58] Spector AC, Grill HJ (1988) Differences in the taste quality of maltose and sucrose in rats: issues involving the generalization of conditioned taste aversions. Chem Senses 13: 95–113.

[59] Loney GC, Torregrossa AM, Carballo C, Eckel LA (2012) Preference for sucralose predicts behavioral responses to sweet and bittersweet tastants. Chem Senses 37: 445–453. 22281530 10.1093/chemse/bjr126 PMC3348172

[60] Finkel T, Holbrook NJ (2000) Oxidants, oxidative stress and the biology of ageing. Nature 408: 239–247. 11089981 10.1038/35041687

[61] Speakman JR, Mitchell SE (2011) Caloric restriction. Mol Aspects Med 32: 159–221. 21840335 10.1016/j.mam.2011.07.001

[62] Campbell AK, Wann KT, Matthews SB (2004) Lactose causes heart arrhythmia in the water flea *Daphnia pulex* . Comp Biochem Physiol Part B: Biochem Mol Biol 139: 225–234. 10.1016/j.cbpc.2004.07.004 15465669

[63] Remmel EJ, Kohmescher N, Larson JH, Hambright KD (2011) An experimental analysis of harmful algae–zooplankton interactions and the ultimate defense. Limnol Oceanogr 56: 461–470.

