# Supplementary material for: Sucralose Induces Biochemical Responses in Daphnia magna
Source: PLoS One. 2014 Apr 3;9(4):e92771. doi: 10.1371/journal.pone.0092771 (PMC3974716; doi:10.1371/journal.pone.0092771)
Supplement: File S1 — This file includes the following: Table S1. Nominal and measured concentrations in the experiment. Table S2. Generalized linear model testing effects of the experimental run. (DOCX) [file pone.0092771.s001.docx]

S1

Sucralose induces biochemical responses in *Daphnia magna*

Ann-Kristin Eriksson Wiklund*, Magaretha Adolfsson-Erici, Birgitta Liewenborg, Elena Gorokhova

Department of Applied Environmental Science (ITM) Stockholm University, SE-106 91 Stockholm, Sweden

*Corresponding author:

Tel. +46 8 674 7251

Fax. +46 8 674 7638

Supporting information

Table S1: Nominal and measured concentrations in the experiments

Table S2. Generalized linear model testing effects.

Table S1. Nominal and measured concentrations in the experiment, nd=not detected.

| Nominal | Sample A | Sample B | | Mean | |
| --- | --- | --- | --- | --- | --- |
| conc. µgL^-1^ |  |  | |  | |
| Control | nd | | nd | | nd |
| 5 | 3.1 | | 3.5 | | 3.3 |
| 50 | 39 | |  | | 39 |
| 500 | 400 | |  | | 400 |
| 5000 | 2700 | | 2900 | | 2800 |

Table S2. Generalized linear model testing effects of the experimental run (experiment), sucralose concentration (LogConc) on the individual protein content in daphnids. Whereas no effect of the sucralose concentration was observed, the animals used in Experiment 1 were significantly larger.

| Regression | Estimate | Standard | Wald | *p* |
| --- | --- | --- | --- | --- |
| Parameter |  | error | statistics |  |
| Intercept | 0.015 | 0.011 | 1.87 | 0.17 |
| Log Conc. | -0.003 | 0.003 | 1.06 | 0,30 |
| Experiment 1 | -0.029 | 0.012 | 5.81 | **0.02** |
| Experiment 2 | -0.001 | 0.014 | 0.009 | 0.92 |
| Experiment 3 | -0.01 | 0.016 | 0.46 | 0.50 |
|  | 2.54 | 2.23 | 1.30 | 0.25 |
